# Simultaneous Bilateral Spontaneous Pneumothorax in an Adult Patient With Pulmonary Langerhans Cell Histiocytosis: A Case Report

**DOI:** 10.1177/2324709618792945

**Published:** 2018-08-06

**Authors:** Vasileios Karamouzos, Christos Prokakis, Fotini Kosmopoulou, Evangelos Karanikolas, Christina Kalogeropoulou, Diamanto Aretha, Nikolaos Panagiotopoulos, Efstratios Koletsis, Dimitrios Velissaris

**Affiliations:** 1 University Hospital of Patras, Rion, Greece; 2 University of Athens, Athens, Greece; 3 University College London Hospitals, London, UK

**Keywords:** lung diseases, interstitial, pulmonary, Langerhans cell, histiocytosis, pneumothorax, bilateral

## Abstract

We report a case of a young female with known history of pulmonary Langerhans cell histiocytosis who was initially presented in the emergency department of a university hospital with respiratory distress. Clinical assessment and diagnostic workup revealed left hemithorax subcutaneous emphysema, bilateral pneumothorax, and atelectasis in both lower lung lobes. The patient was treated with bilateral staged thoracoscopic bullectomy and mechanical abrasion of the parietal pleura combined with chemical pleurodesis with talc. A new occurrence of right-sided pneumothorax was noticed 3 days after surgery, which was treated with chest tube insertion and chemical pleurodesis. The aforementioned surgical approach resulted in complete lung expansion and the patient’s full recovery. A review of pulmonary Langerhans cell histiocytosis and treatment options in cases of pneumothorax due to lung histiocytosis is also presented in this report.

## Introduction

Langerhans cell histiocytosis (LCH) belongs to a group of rare histiocytic disorders characterized by organ infiltration of Langerhans cells causing inflammation and tissue damage. The disease can affect a single organ or can lead to several organ involvement. Episodes of pneumothorax are common in pulmonary LCH (PLCH) patients, but simultaneous bilateral pneumothorax occurs rarely. Furthermore, although respiratory distress due to pneumothorax is a common presentation in the emergency department (ED), clinicians should keep in mind that one presented with spontaneous pneumothorax may conceal an uncommon disease. In this article, we report a case of a 20-year-old woman suffering from PLCH with simultaneous bilateral pneumothorax, treated initially with thoracoscopic bullectomy combined with mechanical abrasion of the parietal pleura and chemical pleurodesis. Relapse of the disease with development of a new pneumothorax was treated with chemical pleurodesis.

## Case Description

A 20-year-old Caucasian female, with a past medical history of hypothyroidism (currently on levothyroxine sodium 50 µg/day) and tobacco use (5 pack-year), presented in the ED of a university hospital in western Greece with acute dyspnea. One year before the current presentation, the patient underwent spirometry, bronchoscopy, chest X-ray, and computed tomography (CT) scan, investigating a persistent unproductive cough. At that time PLCH was diagnosed based on cytology, molecular analysis, and immunohistochemical staining of bronchoscopic material.

**Figure 1.**
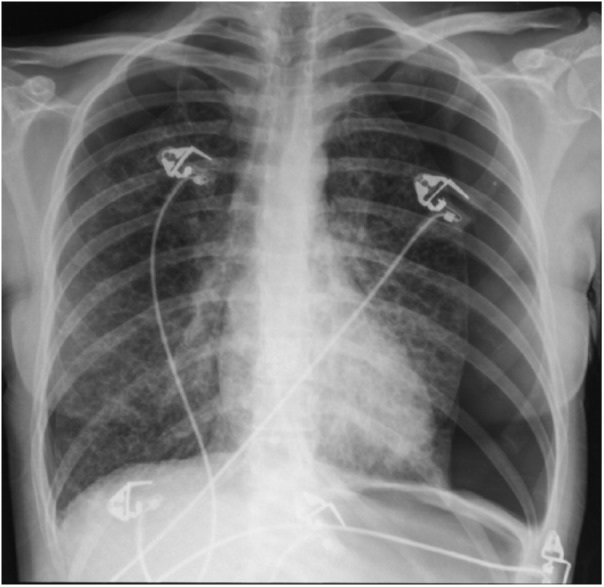
Chest radiograph with bilateral pneumothorax at presentation.

**Figure 2.**
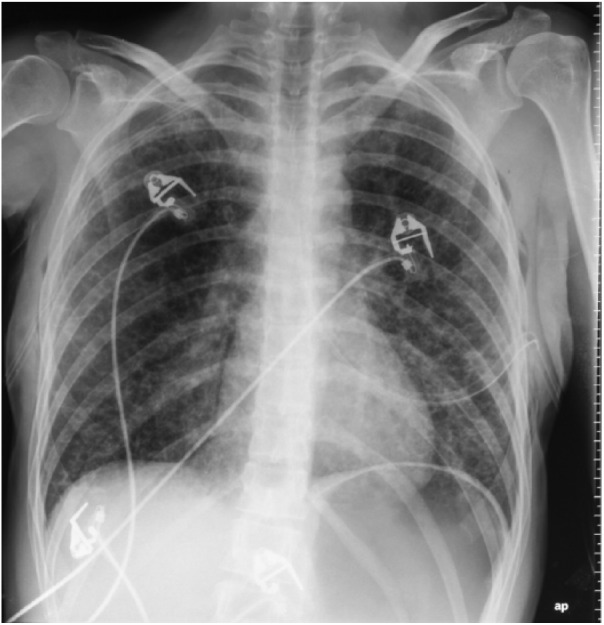
Bilateral lung re-expansion after bilateral drainage tube insertion.

**Figure 3.**
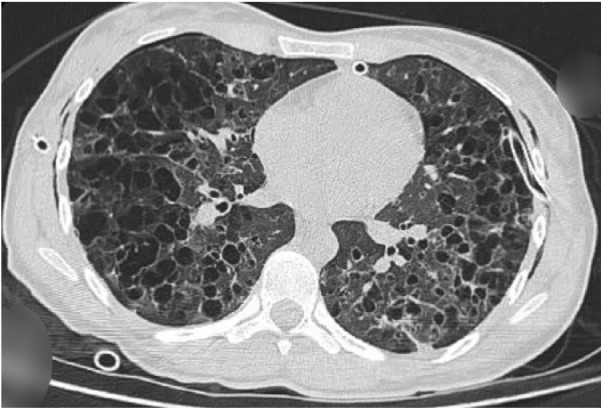
Chest computed tomography scan showing multiple, small, thin-walled, well-defined, rounded cysts that were evenly distributed throughout both lungs.

Initial clinical assessment in the ED revealed respiratory distress with dyspnea, tachypnea (respiratory rate >24 breaths per minute), hypoxemia (PO_2_ = 56 mm Hg on room air), and stable hemodynamic status. Physical examination revealed diminished chest wall movements bilaterally, along with a hyperresonant percussion note bilaterally in the upper and mid zones. Chest auscultation revealed absence of air entry bilaterally in the upper-mid zones and substantially reduced in both the lower zones anteriorly and posteriorly. Furthermore, she had a palpable purpuric rush on the medial surface of the tibia bilaterally. The remainder of the examination was unremarkable. Chest X-ray showed bilateral pneumothorax and intercostal drainage tubes were inserted (Figures 1 and 2). High-resolution computed tomography scan of the chest showed multiple small, thin-walled, well-defined, rounded cysts evenly distributed throughout both lungs (Figure 3), subcutaneous emphysema in the left hemithorax, and atelectasis in both lower lobes. Blood tests results on admission revealed leukocytosis, mild anemia, but no major biochemical abnormalities.

The patient underwent bilateral staged thoracoscopic bullectomy followed by mechanical abrasion of the parietal pleura and chemical pleurodesis with talc. The postoperative course was uneventful. Five days later, chest X-ray showed complete lung expansion. Postoperatively, she was treated with paracetamol (500 mg tds [3 times daily] intravenously [IV]), ampicillin/sulbactam (3 g qds [4 times daily] IV), ranitidine (150 mg bd [twice daily] IV), and levothyroxine sodium (50 µg od [once daily] po [orally]). The patient recovered sufficiently and was discharged from the hospital on postoperative day 5 with advice for smoking cessation and referral to the pulmonology outpatient clinic.

A week later, the patient presented in the ED with mild, right, paravertebral chest pain and respiratory distress (respiratory rate = 23 breaths per minute, PO_2_ = 65 mm Hg on room air). Chest X-ray showed right-sided pneumothorax. An 18-French chest tube was inserted, and the lung was fully re-expanded. Three days later chemical pleurodesis, with tetracycline through the chest tube, was carried out. The chest tube was removed within 24 hours and the lung remained expanded in follow-up chest X-rays. During hospitalization, the patient’s hemodynamic and respiratory status remained stable, laboratory testing did not reveal any abnormalities, and she was discharged to home 4 days later, completely asymptomatic, with radiologic confirmation of complete lung expansion.

## Discussion

LCH is a histiocytic disorder of dendritic cell origin characterized by monoclonal proliferation and infiltration of Langerhans cells to organs.^1^ In the past, due to the diverse phenotypic expression of LCH, several names have been used to describe the disease. Eosinophilic granuloma, Letterer-Siwe disease, Hand-Schuller-Christian syndrome, and Hashimoto-Pritzker syndrome were terms used describing the disease variants. Later the name histiocytosis X was proposed, due to similar histologic findings in the aforementioned diseases while the letter “X” was added to emphasize the “unknown” cell involved. The discovery of Birbeck granules in both lesion cells and epidermal Langerhans cells using electron microscopy led to the term LCH, making all the names used earlier obsolete. Depending on the disease extent, LCH is classified as single- or multi-system, with or without involvement of “risk organs,” such as the liver, spleen, lungs, and the hematopoietic system. PLCH usually occurs as a single-system disease and rarely as part of multi-system involvement.^2^

The exact prevalence of the disease is unknown, mainly due to insufficient epidemiological data. Additionally, patients with mild forms of the disease or experiencing spontaneous remission usually remain undetected. In Europe, a large 5-year study in Belgium reported that 3% of the patients with interstitial disease suffered from PLCH.^3^ In Asia, a study that took place in Japanese hospitals with more than 200 beds reported 160 PLCH cases in 1 year, and finally, in the United States surgical lung biopsy in 502 patients with interstitial disease revealed that 3.4% had PLCH.^4,5^ There is no known genetic predisposition, and the disease affects equally men and women with an incidence peak between the third and the fourth decades of life. Tobacco use is the only epidemiological risk factor that is common in the vast majority of PLCH patients and clearly contributes in the pathogenesis of the disease.^6^

**Figure 4.**
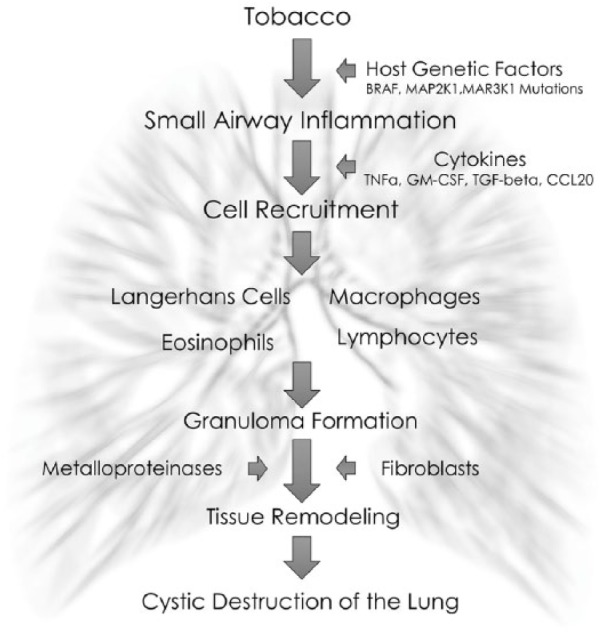
Pulmonary Langerhans cell histiocytosis pathophysiology. BRAF; MAP2K1, mitogen-activated protein 2 kinase 1; MAP3K1, mitogen-activated protein 3 kinase 1; TNFa, tumor necrosis factor alpha; GM-CSF, granulocyte-macrophage colony-stimulating factor; TGF-beta, transforming growth factor beta; CCL20, chemokine (C-C motif) ligand 20.

Initially, inflammatory changes are prominent with Langerhans cells infiltrating the small airways, forming nodules rich in eosinophils, monocytes, macrophages, and lymphocytes that can grow up to 15 mm. Langerhans cells are relatively large with intracytoplasmic Birbeck granules and can be identified with an electron microscope or with positive immunohistochemical staining for S-100, CD1a, and Langering (CD207), which is solely expressed by Langerhans cells. Despite the large number of eosinophils being present in the lesions, peripheral eosinophilia is absent. The disease progresses by gradually destroying the alveoli along with the interstitium and the vascular bed resulting in extensive fibrosis and nodular replacement by cystic lesions (Figure 4) . In the final stages, cystic lesions dominate the middle and upper lobes of the lung to the extent that it may be difficult to distinguish PLCH from advanced emphysema.^7^ Although many hypotheses have been proposed trying to explain the pathophysiology of PLCH, the etiology of the disease still remains elusive.

Langerhans cells are antigen presenting cells that regulate the local immune response and for many years PLCH was thought to be a reactive process. However, recent discoveries demonstrating clonal expansion in PLCH lesions suggest a neoplastic behavior. In many cancer types like melanoma a defect in the MAPK/ERK pathway that regulates cell differentiation, proliferation, and apoptosis can lead to uncontrolled growth of tumor cells. Mutations in genes like BRAF V600E, MAP2K1, and MAP3K1 that encode proteins in the kinase cascade of the MAPK/ERK pathway have been reported in PLCH patients, reinforcing the neoplastic theory.^8-10^

PLCH can be asymptomatic despite the diffuse lung involvement, and the most frequent clinical manifestations in the initial phase of the disease are dry cough and dyspnea on exertion.^11^ Constitutional symptoms such as fever, malaise, and weight loss may be present, and acute chest pain is usually of pleuritic origin. Hemoptysis is rare in PLCH, and when present, excluding other causes, such as malignancy, is mandatory. A common complication of the disease is recurrent spontaneous pneumothorax and on rare occasions life-threatening bilateral pneumothorax may occur. Pulmonary vascular bed is also affected involving arteries and veins, resulting in pulmonary hypertension with increased mortality among patients with PLCH. These vascular changes seem to be independent from parenchymal destruction and no correlation is to date observed between lung function tests and pulmonary hypertension severity.^12^

Pulmonary function tests usually are normal in early disease. Most of the patients have low carbon monoxide diffusing capacity, and in early stages the predominant pattern is restrictive, whereas in advanced stages the obstructive pattern is more common.^13^ Reduced exercise capacity, due to a blend of “pseudorestriction,” air trapping, and gas exchange impairment, is common and with negative impact on quality of life.^14^

The cystic-nodular pattern is the typical radiological feature in PLCH. These lesions are predominant in the upper and middle lung fields, usually sparing the bases,^15^ and high-resolution CT is usually sufficient to establish the diagnosis. Furthermore, fluorine-18-fluorodeoxyglucose positron emission tomography/CT can help determine the activity of PLCH, identify extrapulmonary disease and finally evaluate treatment response.^16^

Prognosis for most of the patients after smoking cessation is relatively good, particularly if long-term lung function tests remain stable. In certain patients, disease progression will remain unaffected by smoking cessation, and although corticosteroid therapy is not supported in general, some case reports have shown benefit from steroid use.^17,18^ Finally, patients with progressive disease may require more aggressive treatment such as lung transplantation.^19^

Approximately 10% of spontaneous pneumothorax cases are caused by diffuse cystic lung diseases like PLCH.^20^ Spontaneous pneumothorax is a recognizable feature of PLCH and results from the destruction of lung parenchyma with associated cystic changes. It is more common in younger adults, with an overall incidence of 11% to 15%. Spontaneous pneumothorax could be the first manifestation of the disease; however, spontaneous bilateral pneumothorax is a rare occurrence and can be fatal.^21^ Recurrent spontaneous pneumothorax necessitates rapid intervention. Therapeutic modalities available are supplemental oxygen, simple aspiration via a catheter, chest tube insertion, pleurodesis, thoracoscopy, video-assisted thoracoscopic surgery, thoracotomy, and pleural surgery.^22,23^

Patients with secondary spontaneous pneumothorax due to PLCH should be referred to surgery early due to the high risk of recurrence after conservative treatment.^24^ Thoracoscopic treatment with mechanical abrasion and chemical pleurodesis is considered the treatment of choice. Although open surgery with parietal pleurectomy may be more effective in achieving pleurodesis, it is usually avoided because of technical difficulties that may ensure during future lung transplantation; however, pleurectomy is not a contraindication to lung transplantation. Bilateral video-assisted thoracoscopic surgery is a safe procedure in the treatment of simultaneous and nonsimultaneous bilateral spontaneous pneumothorax, which reduces also the need for subsequent operations^25^ and allows thoracoscopic bullectomy.^26^ In cases where intensive care support is needed, mechanical ventilation for several days seems to be effective.

## Conclusion

We report our experience with a rare case of pulmonary LCH in a young woman with bilateral spontaneous pneumothorax. Further investigation of these patients is mandatory to exclude potential rare diseases that require multidisciplinary treatment. In these scarce cases, a variety of treatment options should be considered, but tailored therapy based on the specifics of each case is key to successful outcome. The case we are presenting emphasizes the importance of surgical treatment for avoiding recurrence of pneumothorax in patients with LCH and lung involvement.

## References

[1] Favara BE Feller AC Pauli M et al Contemporary classification of histiocytic disorders. The WHO Committee on histiocytic/reticulum cell proliferations. Reclassification Working Group of the Histiocyte Society. Med Pediatr Oncol. 1997;29:157-166. 9212839 10.1002/(sici)1096-911x(199709)29:3<157::aid-mpo1>3.0.co;2-c

[2] Vassallo R Ryu JH Colby TV Hartman T Limper AH. Pulmonary Langerhans’-cell histiocytosis. N Engl J Med. 2000;342:1969-1978. 10877650 10.1056/NEJM200006293422607

[3] Thomeer M Demedts M Vandeurzen K; VRGT Working Group on Interstitial Lung Diseases. Registration of interstitial lung diseases by 20 centres of respiratory medicine in Flanders. Acta Clin Belg. 2001;56:163-172. 11484513 10.1179/acb.2001.026

[4] Watanabe R Tatsumi K Hashimoto S Tamakoshi A Kuriyama T; Respiratory Failure Research Group of Japan. Clinico-epidemiological features of pulmonary histiocytosis X. Intern Med. 2001;40:998-1003. 11688843 10.2169/internalmedicine.40.998

[5] Gaensler EA Carrington CB. Open biopsy for chronic diffuse infiltrative lung disease: clinical, roentgenographic, and physiological correlations in 502 patients. Ann Thorac Surg. 1980;30:411-426. 7436611 10.1016/s0003-4975(10)61291-x

[6] Tazi A. Adult pulmonary Langerhans’ cell histiocytosis. Eur Respir J. 2006;27:1272-1285. 16772390 10.1183/09031936.06.00024004

[7] Roden AC Yi ES. Pulmonary Langerhans cell histiocytosis: an update from the pathologists’ perspective. Arch Pathol Lab Med. 2016;140:230-240. 26927717 10.5858/arpa.2015-0246-RA

[8] Kamionek M Ahmadi Moghaddam P Sakhdari A et al Mutually exclusive extracellular signal-regulated kinase pathway mutations are present in different stages of multi-focal pulmonary Langerhans cell histiocytosis supporting clonal nature of the disease. Histopathology. 2016;69:499-509. 26915300 10.1111/his.12955

[9] Haroche J Cohen-Aubart F Rollins BJ et al Histiocytoses: emerging neoplasia behind inflammation. Lancet Oncol. 2017;18:e113-e125. 28214412 10.1016/S1470-2045(17)30031-1

[10] Radzikowska E. Pulmonary Langerhans’ cell histiocytosis in adults. Adv Respir Med. 2017;85:277-289. 29083024 10.5603/ARM.a2017.0046

[11] Asamoto H Kitaichi M Nagai S Nishimura K Itoh H Izumi T. Pulmonary eosinophilic granuloma—clinical analysis of 17 patients [in Japanese]. Nihon Kyobu Shikkan Gakkai Zasshi. 1995;33:1372-1381. 8821990

[12] Fartoukh M Humbert M Capron F et al Severe pulmonary hypertension in histiocytosis X. Am J Respir Crit Care Med. 2000;161:216-223. 10619823 10.1164/ajrccm.161.1.9807024

[13] Crausman RS Jennings CA Tuder RM Ackerson LM Irvin CG King TE Jr. Pulmonary histiocytosis X: pulmonary function and exercise pathophysiology. Am J Respir Crit Care Med. 1996;153:426-435. 8542154 10.1164/ajrccm.153.1.8542154

[14] Rolland-Debord C Fry S Giovannelli J et al Physiologic determinants of exercise capacity in pulmonary Langerhans cell histiocytosis: a multidimensional analysis. PLoS One. 2017;12:e0170035. 28072848 10.1371/journal.pone.0170035 PMC5225005

[15] Brauner MW Grenier P Mouelhi MM Mompoint D Lenoir S. Pulmonary histiocytosis X: evaluation with high-resolution CT. Radiology. 1989;172:255-258. 2787036 10.1148/radiology.172.1.2787036

[16] Szturz P Řehák Z Koukalová R et al Measuring diffuse metabolic activity on FDG-PET/CT: new method for evaluating Langerhans cell histiocytosis activity in pulmonary parenchyma. Nucl Med Biol. 2012;39:429-436. 22172385 10.1016/j.nucmedbio.2011.10.002

[17] Yokota S Tada S Sugimoto K et al A case of pulmonary eosinophilic granuloma with extrapulmonary involvement treated effectively with steroid hormone [in Japanese]. Nihon Kyobu Shikkan Gakkai Zasshi. 1994;32:78-83. 8114377

[18] Tadokoro A Ishii T Bandoh S Yokomise H Haba R Ishida T. Pulmonary Langerhans cell histiocytosis in a non-smoking Japanese woman [in Japanese]. Nihon Kokyuki Gakkai Zasshi. 2011;49:203-207. 21485154

[19] Suri HS Yi ES Nowakowski GS Vassallo R. Pulmonary Langerhans cell histiocytosis. Orphanet J Rare Dis. 2012;7:16. 22429393 10.1186/1750-1172-7-16 PMC3342091

[20] Cooley J Lee YCG Gupta N. Spontaneous pneumothorax in diffuse cystic lung diseases. Curr Opin Pulm Med. 2017;23:323-333. 28590337 10.1097/MCP.0000000000000391 PMC5563542

[21] Nakhla H Jumbelic M. Sudden death of a patient with pulmonary Langerhans cell histiocytosis. Arch Pathol Lab Med. 2005;129:798-799. 15913433 10.5858/2005-129-798-SDOAPW

[22] Gilligan P Hegarty D Hassan TB. The point of the needle. Occult pneumothorax: a review. Emerg Med J. 2003;20:293-296. 12748158 10.1136/emj.20.3.293 PMC1726083

[23] Sahn SA Heffner JE. Spontaneous pneumothorax. N Engl J Med. 2000;342:868-874. 10727592 10.1056/NEJM200003233421207

[24] Mendez JL Nadrous HF Vassallo R Decker PA Ryu JH. Pneumothorax in pulmonary Langerhans cell histiocytosis. Chest. 2004;125:1028-1032. 15006964 10.1378/chest.125.3.1028

[25] Ayed AK. Bilateral video-assisted thoracoscopic surgery for bilateral spontaneous pneumothorax. Chest. 2002;122:2234-2237. 12475869 10.1378/chest.122.6.2234

[26] Muramatsu T Shimamura M Furuichi M Nishii T Takeshita S Shiono M. Pulmonary Langerhans cell histiocytosis with recurrent pneumothorax. Ann Thorac Surg. 2011;91:e83-e84. 21619953 10.1016/j.athoracsur.2011.01.038

